# Midlife and late-life blood pressure and vascular dementia: a population based observational study

**DOI:** 10.23889/ijpds.v1i1.387

**Published:** 2017-04-19

**Authors:** Mingkai Peng, Guanmin Chen, Karen Tange, Norman Campbell, Eric Smith, Peter Faris, Hude Quan

**Affiliations:** 1 University of Calgary; 2 Alberta Health Services

## Objective

Hypertension and dementia are common disorders in the elderly. The objective of this study is to investigate the association of blood pressure in midlife and late life with the risk of vascular dementia (VaD) using United Kingdom (UK) primary care data.

## Approach

We conducted a retrospective population-based cohort study using The Health Improvement Network (THIN). Two independent study cohorts were created: 1) midlife: individuals aged 60 to 65 with at least 1 blood pressure measurement between the age of 60 to 65; 2) late-life: individuals aged 70 to 75 with at least 1 blood pressure measurements between the age of 70 to 75. The baseline blood pressure for midlife and late-life cohort were categorized into four levels: normal, prehypertension, stage 1, and stage 2 hypertension. Cases of VaD and other risk factors, such as stroke, diabetes, were identified using the Read codes listed in the Quality and Outcome Framework- National Health Science (QOF-NHS). Descriptive statistics were used to compare the demographics and clinical conditions for patients in different blood pressure levels. we used the proportional subdistribution hazard model was used to estimate the association between blood pressure levels with risk of VaD while treating death as a competing risk. Multiple imputation method was used to impute the missing values for the variables of smoking status and Body mass index (BMI) categories.

## Results

In total, there were 265 897 patients (65.1% with stage 1 and 2 hypertension) in the midlife and 211 909 patients (76.3% with stage 1 and 2 hypertension) in the late life cohort, respectively. For midlife cohort, the risk of VaD increased with higher BP levels after adjusting the potential risk confounding factors with a hazard ratio (HR) of 1.15(95% confidence interval 0.88, 1.51) for prehypertension, 1.32(1.01, 1.73) for stage 1 hypertension, 1.33(1.01, 1.73) for stage 2 hypertension; for late-life cohort, after adjusting all the confounding factors, there was no statistically significant association between the risk of VaD and BP levels (HR 1.09 [95% confidence interval 0.86, 1.39] for prehypertension, 1.17(0.92, 1.48) for stage 1 hypertension, and 1.11(0.88, 1.42) for stage 2 hypertension). 

## Conclusion

Our study show high blood pressure in midlife is a significant risk for vascular dementia in the late-life. Given the strength and consistency of this evidence, greater effort should be placed for early diagnosis of hypertension and tight control of blood pressure for hypertensive patients in the prevention of vascular dementia.

